# Historico-Pathological Researches, Being Contributions to the History of Epidemics

**Published:** 1841-04

**Authors:** 


Art. XIII.—*Historisch-pathologische Untersuchungen, als beiträge zur geschichte der Volkskrankheiten.* Von Dr. H. Haeser.—*Dresden*, 1839. pp. 329.

*Historico-pathological Researches, being contributions to the History of Epidemics.* By Dr. H. Haeser.—*Dresden*, 1839.

This work has enjoyed much reputation upon the continent, but it is needless now to do more than notice our having received it; for in a former Number of our Journal (vol. VII, p. 120,) we reviewed at some length Dr. Jahn's new system of pathology, upon which Dr. Haeser's work is professedly founded. "Diseases," says our author, "obey no less certain and determinate laws than those which govern every other living being. Paracelsus and others suspected this fact; but by no one has it been so fully illustrated as by Jahn." Founding his opinions upon the theories of Jahn, with which our readers are too well acquainted to warrant our again detailing them, Dr. Haeser has traced with great industry and praiseworthy research, the history of the epidemics of the past and present ages; so far the volume is one of much merit; but it contains nothing new, either in doctrine or in practice.

